# Medicolegal Causation Investigation of Bacterial Endocarditis Associated with an Oral Surgery Practice Using the INFERENCE Approach

**DOI:** 10.3390/ijerph18147530

**Published:** 2021-07-15

**Authors:** Putri Dianita Ika Meilia, Maurice P. Zeegers, Herkutanto, Michael D. Freeman

**Affiliations:** 1 Care and Public Health Research Institute (CAPHRI), Maastricht University Medical Center+, Universiteitssingel 40, 6229 ER Maastricht, The Netherlands; m.zeegers@maastrichtuniversity.nl (M.P.Z.); m.freeman@maastrichtuniversity.nl (M.D.F.); 2 Department of Forensic Medicine and Medicolegal Studies, Faculty of Medicine, University of Indonesia, Jl. Salemba Raya No. 4, Salemba, Jakarta Pusat 10430, Indonesia; herkutanto@yahoo.co.id

**Keywords:** INFERENCE approach, malpractice litigation, medicolegal causal analysis, quantification of causation, bacterial endocarditis, infection prevention and control practices

## Abstract

Investigating causation is a primary goal in forensic/legal medicine, aiming to establish the connection between an unlawful/negligent act and an adverse outcome. In malpractice litigation involving a healthcare-associated infection due to a failure of infection prevention and control practices, the medicolegal causal analysis needs to quantify the individual causal probabilities to meet the evidentiary requirements of the court. In this paper, we present the investigation of the most probable cause of bacterial endocarditis in a patient who underwent an invasive procedure at a dental/oral surgical practice where an outbreak of bacterial endocarditis had already been identified by the state Department of Health. We assessed the probability that the patient’s endocarditis was part of the outbreak versus that it was an unrelated sporadic infection using the INFERENCE (Integration of Forensic Epidemiology and the Rigorous Evaluation of Causation Elements) approach to medicolegal causation analysis. This paper describes the step-by-step application of the INFERENCE approach to demonstrate its utility in quantifying the probability of causation. The use of INFERENCE provides the court with an evidence-based, transparent, and reliable guide to determine liability, causation, and damages.

## 1. Introduction

Causation is broadly defined as the cause-and-effect relationship between an action and an outcome. The investigation of causation is a primary goal in forensic/legal medicine, a multifaceted discipline that is a hybrid of medicine, law, and public health [1,2]. Medicolegal analysis of causation is necessary in legal actions involving injury, disease, and death to establish the nexus between an unlawful or negligent act and a subsequent adverse outcome. Depending on the circumstances, the complexity of causal evaluations ranges widely, with many assessments of simple cases requiring only the application of scientific common sense and professional intuition [3]. In more complex cases, however, a more in-depth causal investigation is warranted. A representative example is a medical negligence (i.e., malpractice) action involving a disease outbreak that has allegedly resulted from a faulty medical procedure or practice. There are many facets to such an investigation, and a systematic approach that maximizes the transparency of methods is ideal.

Healthcare-associated infections (HAIs) arise in several scenarios [3,4]. Most infections reflect the largely unpreventable background rate of HAIs resulting from the abundance of pathogens found in healthcare settings. A smaller number of infections are preventable and sporadic, such as peritonitis due to an overlooked bowel injury during surgery. An even smaller group of preventable infections occurs in clusters or outbreaks and results from the repeated failure of infection prevention and control (IPAC) practices.

HAIs can occur in practically every healthcare setting, including dental and oral surgical practices [5,6,7]. A particularly serious HAI associated with some dental procedures is bacterial endocarditis (BE). In dental practice, there is a well-recognized risk of bacteremia by oral commensal microorganisms via disruption of the oral mucosa or gums [8,9,10]. Bacteremia is, in turn, associated with potentially severe and life-threatening complications, including BE, an infection of the lining of the heart, including the heart valves [11,12].

An infectious disease outbreak is usually defined as an increase of infections compared to the expected number during a specific time and in a certain place [13,14]. If, in a healthcare setting, it can be demonstrated that the increase is not due to known external factors, then it is reasonable to infer that a systematic failure of IPAC is the most logical explanation for the infections.

Initial investigations of HAI outbreaks are ordinarily conducted by public health agencies with the legal authority to inspect premises and practices. When a subsequent legal action for an individual personal injury is associated with an HAI outbreak, the methods for medicolegal analysis will likely incorporate the public health agency findings and methods but also require unique additional steps to quantify the *individual/specific* causal probabilities for the judge or jury to meet the evidentiary requirements of the court [15].

This paper presents the investigation of the most probable cause of BE in a patient who underwent an invasive procedure at a dental/oral surgical practice where an outbreak of BE had already been identified through a public health investigation by the state Department of Health [16]. The purpose of the medicolegal investigation was to assess the probability that the patient’s BE was related to the dental procedure (i.e., that the patient’s infection was part of the outbreak) versus the probability that it was an unrelated sporadic infection. The INFERENCE (Integration of Forensic Epidemiology and the Rigorous Evaluation of Causation Elements) approach to medicolegal causation analysis was used to organize the evidence and quantify the causal probabilities for presentation as expert opinion in a personal injury civil action brought by the patient [17].

## 2. Materials and Methods

### 2.1. Case Description

The patient, “Mrs. D”, was a 69-year-old female in generally good health who underwent a procedure at “Dr. X’s” dental and oral surgery practice.

### 2.2. Relevant Medical History

A chronology of Mrs. D’s relevant medical history is detailed in Table 1.

### 2.3. Public Health Investigation Findings

An investigative team from the state Department of Health conducted two unannounced inspections and environmental assessments of the clinic [16]. The investigation revealed that Dr. X performed all procedures at the practice with at least one assistant. The initial assessment identified multiple breaches of IPAC practices from the Centers for Disease Control and Prevention (CDC)’s Guidelines for Infection Control in Dental Healthcare Settings [6].

A total of 3756 unique patients were treated in the clinic during 2013 and 2014. Patients of the practice who were evaluated in an emergency department or hospitalized from 1 January 2013 through 30 June 2015 were identified. The diagnostic codes associated with their emergency department visit or hospitalization were reviewed. The public health investigation identified 14 confirmed cases/patients with *E. faecalis* endocarditis [16]. All patients had surgery at the same clinic performed by Dr. X. 

Table 2 lists the illness onset versus procedure timing and other key features of the 14 patients. The patients are listed in chronological order by the date of the oral procedure.

### 2.4. Legal Allegations

Mrs. D filed a suit against Dr. X’s practice, alleging that she contracted BE because of negligent IPAC violations occurring during her oral surgery. The defense argued that most cases of BE, and infective endocarditis in general, do not occur in dental practices but occur sporadically or are idiopathic [11,12,18,19]. Thus, while Mrs. D’s BE *may* have resulted from her exposure to Dr. X’s practice, it also could have arisen spontaneously. Therefore, it could not be said that the infection resulted from the exposure to the standard required by the court, which was “on a more probable than not (>50% probable) basis.”

### 2.5. Causation Analysis

The case was analyzed using the INFERENCE framework. INFERENCE consists of two main parts, i.e., (1) the definition of the relevant medicolegal question and terms and (2) a three-step process of causal analysis based on the Hill criteria (plausibility, temporality, and alternative explanations), which is designed to quantify the probability of causation.

In Mrs. D’s legal action, the use of INFERENCE was necessitated by the defendant’s theory that it was only *possible* that her BE was caused by Dr. X’s negligence. Therefore, it was necessary to compare/quantify how much more probable it was that Mrs. D acquired her BE due to the dental procedure performed by Dr. X, versus her chance of contracting the infection if she had not had the procedure.

## 3. Results

### 3.1. Steps of the INFERENCE Approach, Applied to the Specific Facts of Mrs. D’s BE Infection

#### 3.1.1. Step 1—Define the Medicolegal Causation Question and Determine Whether INFERENCE Is Required

The causal question was formulated based on the competing causal theories/legal hypotheses of the opposing parties. This causal question was in the form of comparison or ratio of probabilities based on counterfactual reasoning, as follows: “What was the risk of BE to Mrs. D as a patient of Dr. X during the timeframe of interest compared to her risk of BE during the same timeframe had she not been a patient of Dr. X?” The complexity of the causal question and, in particular, the need for a quantified answer ruled out the use of intuition alone as a sufficient approach. Hence, the analysis proceeded to the next step.

#### 3.1.2. Step 2—Evaluate the Relevant Factual Basis for the INFERENCE Causal Approach

The available evidence relevant to the causal question was assessed in this step, including the demographic, medical, and historical information. At 69 years of age, Mrs. D belonged to the age group with the highest all-cause risk of infective endocarditis [18,19,20]. The timing of her symptoms and diagnoses were detailed and confirmed from her medical records. Although it was not until more than four months after her dental surgery that Mrs. D was diagnosed with BE, she began experiencing signs and symptoms consistent with symptoms of bacteremia after approximately 20 days post-procedure, including fever, flu-like symptoms, radiating pain, and dizziness [21]. Mrs. D had no known predisposing factors to BE during this 20-day interval, e.g., intravenous drug use, congenital heart diseases, and chronic intravenous access. These relevant demographic and medical data, the issue of appropriate timing and sequence, and the absence of possible alternative explanations were indicative of causation according to Hill’s criteria [22].

#### 3.1.3. Step 3—Identify the Medicolegal Causation Elements for Calculation of the PC

The medicolegal causation question was then broken down into the elements required to calculate the probability of causation [17,23]:

- The condition of interest: enterococcal BE.
- The alleged primary harmful exposure resulting in the condition of interest: the dental surgical procedure provided by Dr. X, in which IPAC was allegedly violated.
- The hazard period: the time between the primary harmful exposure and first history of symptomatic manifestation of the condition of interest, i.e., 20 days.
- Potential competing causes: enterococcal BE risk resulting from all known and unknown causes, aside from the primary harmful exposure, occurring during the 20-day hazard period (see Table 2). Because there are no competing events or predictive factors that put Mrs. D at greater risk than the average woman in her age group, her risk is equal to the hazard-period-adjusted background rate of BE relevant to the population group of females aged ≥ 60 years.

#### 3.1.4. Step 4—Calculate the Comparative Risk Ratio and Probability of Causation

In this step, the primary harmful exposure and plausible competing cause risks were assessed, quantified, and compared as a ratio. First, the plausibility of the suspected primary harmful exposure and competing risks were assessed as causes of the condition of interest. Then, the risk of the condition of interest given the primary harmful exposure was compared with the risk of the condition of interest from the alternative causes as a ratio (i.e., comparative risk ratio or CRR). These elements of the final step, as applied to the facts of Mrs. B’s infection, were as follows:

*Plausibility assessment*: The available facts indicated that the recent invasive dental procedure, advancing age, and possible associated degenerative valvular disease were well established as plausible causes of Mrs. D’s BE [9,11,12,19,20].

*Comparative risk ratio calculation:* To proceed to this final step, the numerator (risk due to the primary harmful exposure) and denominator (risk due to alternative cause) values of the comparative risk ratio needed to first be estimated. The risk of the condition of interest given the primary harmful exposure is the number of people who contracted BE among Dr. X’s patients during the timeframe of interest, which was 0.37%. This figure was calculated from the information that 14 cases of BE were diagnosed among 3756 patients who attended the practice in 2013–2014. For ease of communication to laypersons, the frequency can also be described as 1 case in 268 patients.

The CRR denominator value is an estimate of the hazard-period-adjusted risk of the condition of interest due to all other plausible alternative causes. For Mrs. D’s case, this value was estimated from the annual risk of enterococcal endocarditis for women in her age group (i.e., ≥60 years) living in the U.S. The U.S. national hospital data indicate that there were 1255 cases of enterococcal endocarditis in women ≥ 60 years old during 2013–2014, among a total population of approximately 32,000,000 women aged 60 years and above [24,25]. This frequency equates to an annual incidence of 0.00196%, or a base rate of 1 case per 51,020 per year. This annual base rate risk was adjusted to the hazard period to arrive at a 20-day cumulative risk by dividing the annual risk by 18.25 (as there are 18.25 20-day periods in a year), resulting in a final estimate of the risk that Mrs. D would have acquired BE during the 20-day hazard period in the absence of the primary harmful exposure of 1 in 931,115.

The comparative risk ratio (*CRR*) was thus calculated as follows:

(1) $$CRR=\frac{1\;\mathrm{in}\;268}{1\;\mathrm{in}\;931,115}≌3474$$

The comparative risk ratio indicates that Mrs. D’s BE was approximately 3474 times more likely to be caused by her oral surgery than due to any known (and unknown) competing cause.

*Probability of causation (PC) calculation*: To allow for easier comparison to the standard of proof as required by the court, the comparative risk ratio was converted into a probability of causation using the following formula:

(2) $$\mathrm{PC}\;=\;\frac{\left(CRR-1\right)}{CRR}\;\times 100\%\;=\;\frac{\left(3474-1\right)}{3474}\;\times 100\%\;≌\;99.9\%$$

The calculation shows a greater than 99.9% probability that Mrs. D’s BE was causally related to the oral surgery at Dr. X’s practice.

### 3.2. Flowchart of the INFERENCE Process

**Figure 1:**
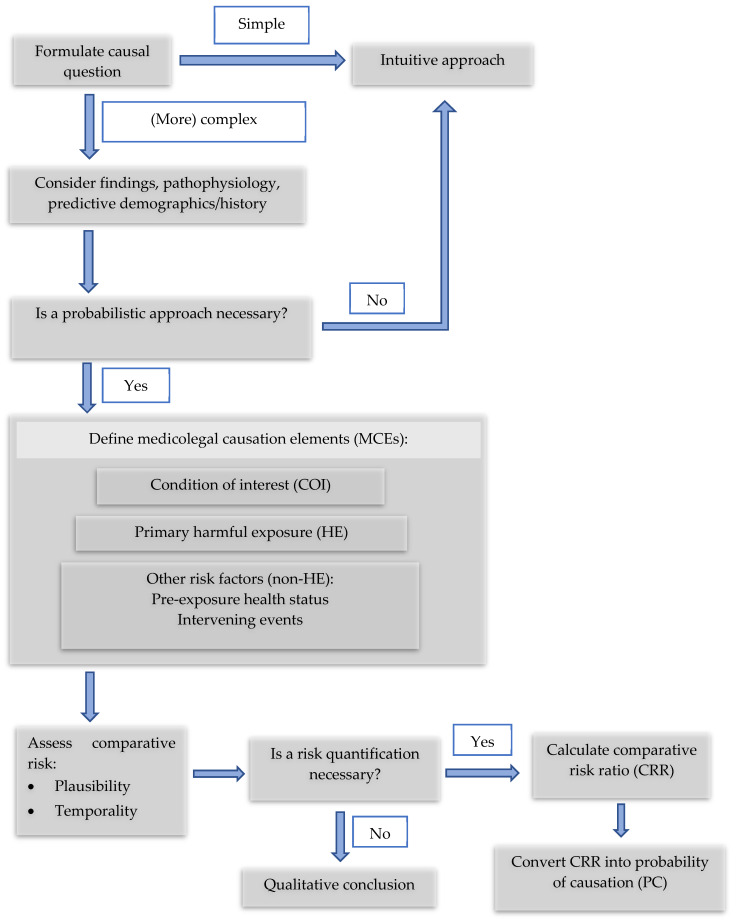
Flowchart of the INFERENCE approach [17].

Flowchart (Figure 1) of the INFERENCE Process:

## 4. Discussion

This paper aims to describe the step-by-step application of the INFERENCE approach [17] to a medical negligence case. The case described in this report is used as an example to quantify the probability that a case of BE was part of an outbreak related to a single clinic, as opposed to a sporadic and unrelated case.

Causality is an unobservable phenomenon and, thus, can only be inferred retrospectively via comparison of risks. This fact results in the inability to prove or demonstrate causation with direct evidence, unlike a clinical diagnosis [26]. Due to this reason, the defense could raise the theory that the plaintiff’s infection was just as likely to have been a sporadic case of BE of unknown origin as a competing theory to the plaintiff’s attribution of the BE to negligently delivered oral surgery care. While there is no way to know precisely where all of the infections occurred, the magnitude of increased risk to patients of Dr. X cannot be explained by random accumulation of infections that are coincidental to the exposure at the practice.

The application of the INFERENCE approach provided a series of steps and instructions that resulted in quantification and comparison of the probabilities associated with the competing theories. One advantage of the approach was that the court was presented with a probability of causation that could be compared to the required standard of proof, i.e., that Mrs. D’s BE was more likely related to the outbreak than not.

Without a quantified causation opinion, the court would have had to choose between well-qualified and persuasive experts from both sides, who both claim to have followed generally accepted scientific methods but still came to contradictory opinions regarding the most probable cause of Mrs. D’s infection. The use of INFERENCE provides the court with a guide for the degree of weight that should be assigned to the defense’s causation theory (i.e., <0.1%) in making determinations of liability, causation, and damages.

Legal standards and customary methods used for causal analysis may differ in various countries and jurisdictions. Thus, one of the reasons for the present study is to describe a method that is meant to be used universally, when appropriate. We have developed INFERENCE as a cognitive approach for the medicolegal/forensic medical practitioner that can be used in various settings, regardless of the legal system.

It is worth noting that the denominator value for the comparative risk ratio calculation (i.e., the base rate risk of BE due to causes other than the oral surgery exposure) was intentionally overestimated. Mrs. D had no risk factors for BE, and thus using the rate for the entire population likely underestimated the risk for the subpopulation with BE risk factors (e.g., artificial heart valves or intravenous drug use) and overestimated it for most of the population, who, like Mrs. D, have no BE risk factors. The choice to overestimate the base rate of BE and, thus, inflate the denominator to some degree was deemed appropriate based on a “safety analysis” approach that minimizes Type I (false positive) error risk at the expense of an increased Type II (false negative) error risk. Thus, to the degree that bias exists in the analysis, it favors the opposing (defense) rather than the retaining (plaintiff) party. The large comparative risk ratio value, however, made the issue moot.

## 5. Conclusions

The INFERENCE approach is a practical stepwise system for organizing evidence and quantifying causal probabilities when causation is disputed. Despite some increased complexity, a key advantage of an INFERENCE analysis is that it provides the court with an evidence-based, transparent, and reliable expert quantification of the probability of causation, as demonstrated in the above analysis.

## Figures and Tables

**Table 1:** Relevant medical history timeline for Mrs. D. Signs and symptoms associated with bacterial endocarditis (BE) are underlined.

Time (Relative to Procedure)	Evidence
Day 0	Mrs. D underwent a lumpectomy on the lateral tongue and side of the cheek performed by Dr. X under IV sedation. Post-procedure, Dr. X prescribed an antibiotic (azithromycin).
Day 2	Mrs. D noticed the left side of her neck was swollen, and by the following day, she had pain in her ear and beneath the angle of her left jaw.
Day 4	Mrs. D contacted Dr. X’s office with a complaint of a swollen gland and was told to take an over-the-counter non-steroidal anti-inflammatory drug (NSAID).
Day 14	Mrs. D returned to Dr. X for follow-up and was advised that the excised mass was benign. The incision was healing well. Further follow-up was scheduled in 4 days for the swollen glands, which Mrs. D canceled as she was feeling better.
Day 20	Mrs. D awoke with a fever and dizziness. She managed the fever with ibuprofen but continued to feel unwell over the next two weeks.
Day 49	Mrs. D presented to an internal medicine specialist reporting flu-like symptoms and left shoulder pain. Cardiac auscultation revealed a regular rate and rhythm without a murmur.
Day 63	Mrs. D awoke with severe pain radiating from the left shoulder to the breastbone, with dorsal muscle spasms. The pain persisted in her lower back and left scapula.
Day 80	Mrs. D began spiking fevers again. She continued to feel unwell, dizzy, and weak, and developed a productive cough.
Day 90	She revisited the internist who diagnosed acute bronchitis and was prescribed a 10-day course of levofloxacin 500 mg.
Day 118	Mrs. D continued to feel unwell. The internist refilled the levofloxacin and referred Mrs. D for infectious diseases consult due to continuous fever (39.4 °C), sweats, and dizziness.
Day 128	Mrs. D presented to the emergency department with spiking fevers and an altered mental state. She began urgent workup for fever of unknown origin including blood and urine cultures, electrocardiography (ECG), and echocardiogram and commenced empirical vancomycin and ceftriaxone. She demonstrated signs of edema in both lower extremities. The ECG showed sinus tachycardia (108 bpm) with frequent premature ventricular contractions. The results of the blood cultures revealed Gram-positive cocci and *E. faecalis*. Initial transesophageal echocardiogram revealed a possibly prolapsed aortic valve, highly suspicious for vegetation. Thoracic magnetic resonance imaging (MRI) demonstrated cardiomegaly with left atrial enlargement and small bilateral pleural effusions.
Day 133	Repeat echocardiogram confirmed signs of aortic valve endocarditis, a large mobile vegetation with likely perforation of aortic leaflets, and moderate to severe aortic regurgitation. Mrs. D was diagnosed with acute aortic valve endocarditis with valve perforation resulting in dynamic shortness of breath, likely secondary to congestive heart failure. She required urgent surgery.

**Table 2:** List of age, sex, date of surgery, time between surgery and symptom onset (days), and time between surgery and diagnosis of bacteremia of the cases. **Mrs. D is Patient #13**.

Patient #	Age at Surgery	Sex	Date of Surgery	Days between Surgery and Symptom Onset (Hazard Period/HP)	Days between Surgery and Diagnosis of *Enterococcus faecalis* Bacteremia
01	20	M	18/01/2013	2	104
02	54	M	23/01/2013	33	115
03	17	M	04/04/2013	72	79
04	46	F	19/04/2013	30	48
05	54	M	03/06/2013	73	149
06	18	F	08/07/2013	14	59
07	77	M	27/08/2013	10	35
08	65	F	25/09/2013	1	30
09	16	M	07/05/2014	54	147
10	29	M	20/06/2014	13	75
11	23	M	27/06/2014	32	110
12	21	M	18/07/2014	Unknown	90
13	69	F	31/07/2014	20	128
14	49	M	21/08/2014	8	77

## Data Availability

Not applicable.
